# Background and roles: myosin in autoimmune diseases

**DOI:** 10.3389/fcell.2023.1220672

**Published:** 2023-08-23

**Authors:** Longsheng Fu, Yonghui Zou, Boyang Yu, Daojun Hong, Teng Guan, Jinfang Hu, Yi Xu, Yaoqi Wu, Junping Kou, Yanni Lv

**Affiliations:** ^1^ Department of Pharmacy, The First Affiliated Hospital of Nanchang University, Nanchang, Jiangxi, China; ^2^ Jiangsu Key Laboratory of TCM Evaluation and Translational Research, Department of Pharmacology of Chinese Materia Medica, School of Traditional Chinese Pharmacy, China Pharmaceutical University, Nanjing, Jiangxi, China; ^3^ Department of Neurology, The First Affiliated Hospital of Nanchang University, Nanchang, Jiangxi, China; ^4^ Department of Human Anatomy and Cell Science, University of Manitoba, Winnipeg, MB, Canada

**Keywords:** myosin, autoimmune, autoimmune myocardioptis, NMMHC IIA, multiple sclerosis, Alzheimer, Parkinson

## Abstract

The myosin superfamily is a group of molecular motors. Autoimmune diseases are characterized by dysregulation or deficiency of the immune tolerance mechanism, resulting in an immune response to the human body itself. The link between myosin and autoimmune diseases is much more complex than scientists had hoped. Myosin itself immunization can induce experimental autoimmune diseases of animals, and myosins were abnormally expressed in a number of autoimmune diseases. Additionally, myosin takes part in the pathological process of multiple sclerosis, Alzheimer’s disease, Parkinson’s disease, autoimmune myocarditis, myositis, hemopathy, inclusion body diseases, etc. However, research on myosin and its involvement in the occurrence and development of diseases is still in its infancy, and the underlying pathological mechanisms are not well understood. We can reasonably predict that myosin might play a role in new treatments of autoimmune diseases.

## 1 Introduction

The myosin superfamily is a group of molecular motors moving along a linear actin axis. Myosins were originally found when Kuehne observed a skeletal muscle contraction in 1864 and they were named after Roman numerals in 35 classifications (Kull, 2000). Myosin plays a dominant role in living things, and the influence of the working mechanism has been extensively researched, which not only helps people better understand how the body converts chemical energy into mechanical energy but also provides a good reference for the design of molecular machinery (Takaki et al., 2022). For example, with the deepening of myosin II research, diseases such as myocarditis could be explained by genetic means.

In normal physiological conditions, the immune system does not act in response to the human body itself (self-tolerance). Autoimmune diseases are characterized by dysregulation or deficiency of the immune tolerance mechanism, inducing an immune response to the human body itself (Coss et al., 2022; Johnson and Jiang, 2022). Autoimmune diseases are a serious health hazard due to organic damage and dysfunction and are characterized by the excessive activation of T and B lymphocytes, and autoantibodies (Mageau et al., 2023). The etiology is obscure, and the appropriate treatment is debatable. Glucocorticoids and traditional antirheumatic drugs are the most commonly used drugs, having positive effects on attenuating inflammation, analgesia, and ameliorating or delaying disease progression. In addition, other potential treatment options, such as stem cell transplantation, biologics, or new plant agents, might be considered for those who respond poorly or cannot tolerate first-line therapy (Dang et al., 2020). Although autoimmune diseases cannot be completely cured, treatments can keep symptoms at bay and improve the quality of life for those who have already developed symptoms. Based on previous research findings, we have discovered that the link between myosin and autoimmune diseases is much more complex than we had hoped. Myosins were abnormally expressed in a number of autoimmune diseases, and participates in a complicated pathological process of autoimmune diseases (Zhang et al., 2020a), but the reported literature on this topic is very limited. Although the available literature is scarce, we have reasons to believe that a breakthrough can be made in the relation between myosin and autoimmune diseases. This review fully describes the implications of myosin in autoimmune diseases.

**FIGURE 1:**
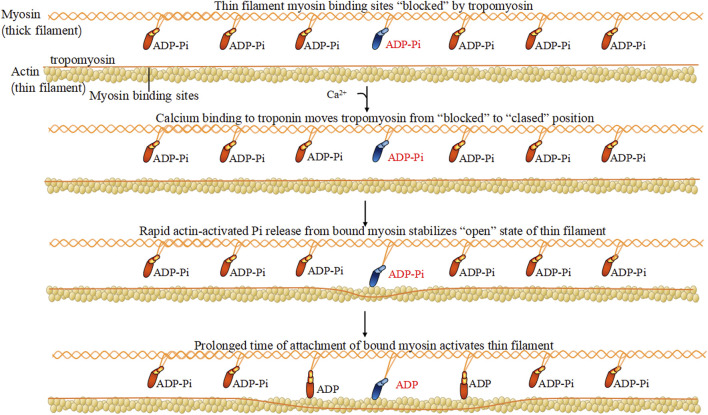
Myosin hydrolyzes ATP.

Myosin has long mismatched dimers and a Y structure of ∼160 nm. Under a scanning electronic microscope, the myosin structure may be observed, including the entire set of long peptide chains and two pairs of short peptide chains, constructing two spherical heads and a long rod-shaped tail. Myosin was first found in muscular tissue, and since 1970, scientists have identified more myosins in mice and humans. Each myosin is constructed of a common set of three standard parts, including the head, neck, and tail, and a composition of heavy and light chains. After 1970, many non-muscle myosins were gradually discovered, there are 35 members in the superfamily. Myosin within the cytoskeleton, as its “molecular motor,” binds actin called acto-myosin and effectively converts ATP (Triphosadenine) energy into muscle contractions. The most important feature for heavy chains is an energy-producing motor to enable their metabolic capacity. The head part of the heavy chain of myosin has the most conservative structure, generating energy and momentum via the binding affinity of actin and the ATP binding site for the ligand (Figure 1). Regulatory light chains dedicated to certain functions have myosin regulatory enforcement functions, and the phosphorylation/dephosphorylation state has a large impact on the activity of myosin. Based on the earliest and most sliding filament theory (Spudich, 2001; Powers et al., 2021), combined with the later developed swinging lever arm model explains how the myosin combines with F-actin during the process of filament sliding (Marcucci et al., 2021).

## 2 Myosins involvement in autoimmune diseases

### 2.1 Myosin immunization modeling experiment

In a mouse model, cardiac myosin is an autoantigen, especially in the later phases of postinfectious myocarditis. Heart-reactive antibodies were identified as cardiac isoforms of myosin due to autoimmunization (Rose et al., 1987). The application of emulsified cardiac myosin itself could establish an experimental autoimmune myocarditis model (Lee et al., 2016). Cardiac myosin could stimulate the production of heart-specific antibodies accompanied by cardiac muscle striations and sarcolemma (Rose et al., 1987). In cardiac myosin-immunized experimental autoimmune myocarditis mice, activated extrathymic T lymphocytes expressed high levels of LFA-1 and IL-2R (Interleukin 2 receptor) beta-chains while inducing differentiated CD4-CD8- T cell movement to the sites of the cardiac lesion (Hanawa et al., 1993). It has also been reported that myosin immunization can induce experimental autoimmune myositis in guinea pigs (Chen et al., 2018).

### 2.2 The molecular mechanism of myosins on cell of the immune system

#### 2.2.1 Contraction dysfunction

**FIGURE 2:**
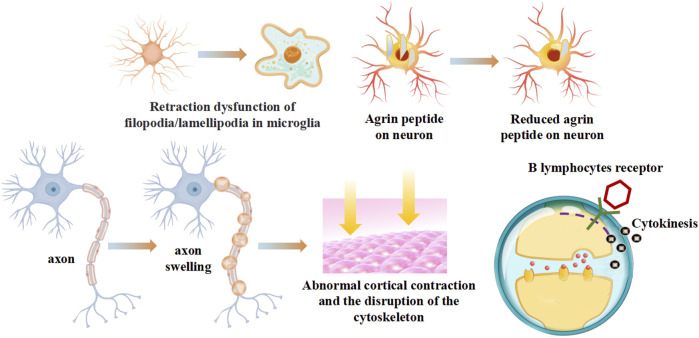
The contraction dysfunction of myosins on autoimmune diseases.

One of the chief effects of myosins on the cell of the immune system could lead to the contraction dysfunction of autoimmune diseases. The myosin motor operates as a linear motor (Chatterjee et al., 2022), vibrating along a linear axis. Studying the structure of the motor enables us to understand its mechanism of directed movement (Reconditi et al., 2011). Based on the classic swinging cross bridge model (Spudich, 2001), when ATP hydrolysis occurs, the head of myosin leans toward F-actin, and deviates from actin to trigger the movement of thin filaments. Then the motor head moves away from F-actin, participates in a muscle contraction. The axon shape change of myosin induces active cortex contraction, promoting the disruption of the cytoskeleton (Riccobelli, 2021) (Figure 2). Or the retraction dysfunction of filopodia/lamellipodia could be induced by actin/myosin-based contractions damage (Reichert and Rotshenker, 2019). The rod-like morphology aggregated by the myosin impaired the junctions between neuromuscular (O’Connor et al., 2018). Myosin IIA has been identified as a negative regulator of B lymphocytes activation. B lymphocytes response are thought to depend on contractile activity of non-muscle myosin IIA. Deletion of the myosin IIA heavy chain reduced serum immunoglobulin levels and no strong antibody response was produced during immunization (Hoogeboom et al., 2018) (Figure 2).

#### 2.2.2 Regulatory myosin light chains related mechanism

**FIGURE 3:**
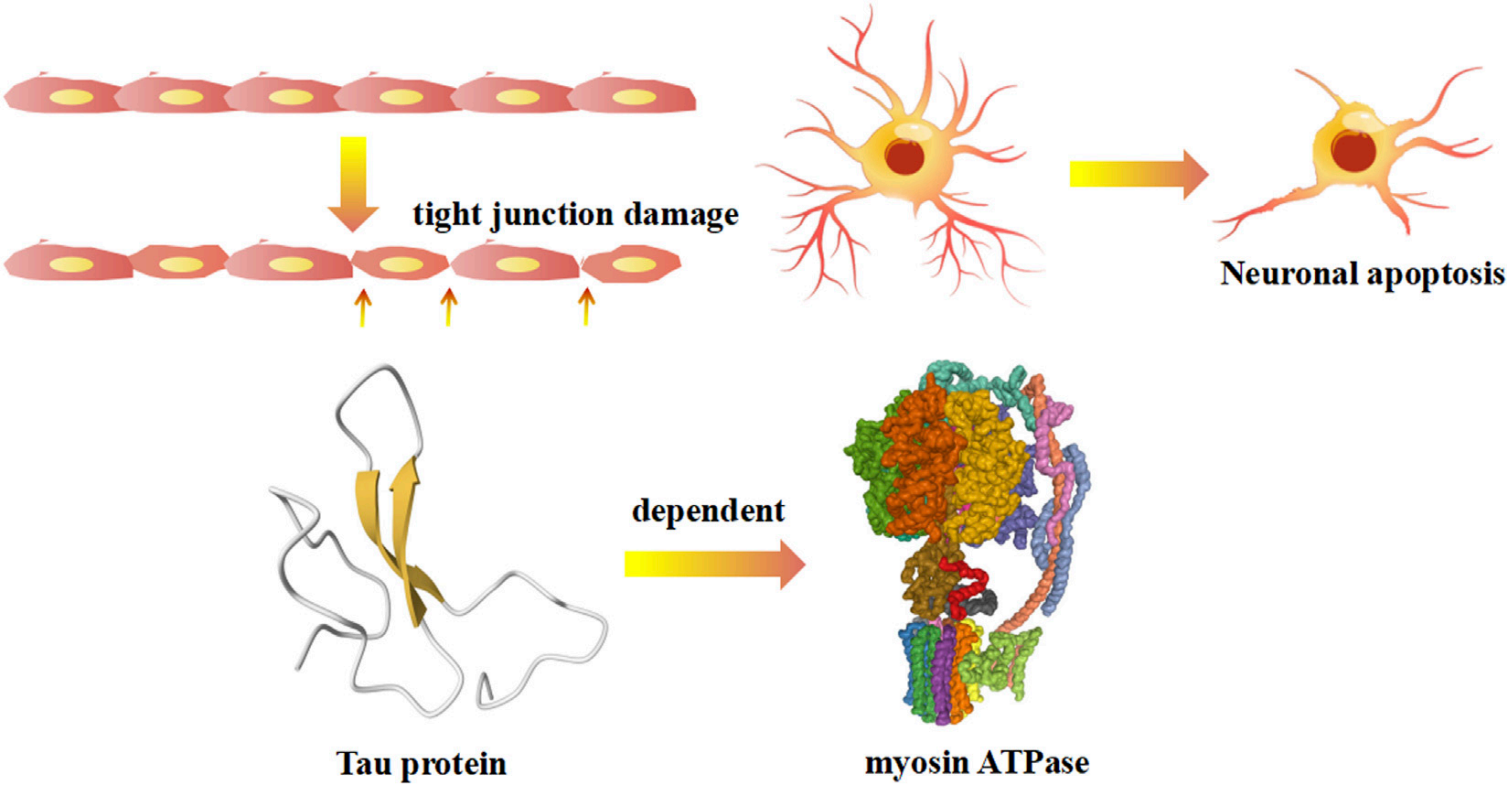
The regulatory myosin light chains related mechanism on autoimmune diseases.

Regulatory light chains dedicated to certain functions have myosin regulatory enforcement functions, and the phosphorylation/dephosphorylation state has a large impact on the activity of myosin. The myosin heavy chain has ATP enzyme vitality, and the myosin light chain can be activated by phosphorylation of Ca^2+^-CaM (calmodulin), causing conformation transitions (Takaki et al., 2022) (Figure 3). There are two light chains with their own position on the heavy chain: one is the regulatory light chain 2 (MLC2), and one is the essential light chain (MLC1). Under normal physiological conditions, the phosphorylation of MLC2 stand at 40% overall. The abnormal phosphorylation is the pathological base and associated factors of diseases. Terminal of MLC2 have two phosphorylation site: serine and threonine residues. After phosphorylation, the MLC2 form transforms from a compressed form to end as a extended form. Phosphorylated MLC2 can increase the mobility of myosin transbridge and promote the movement of motor head. In addition, phosphorylated MLC2 can also accelerate the release of phosphoric acid, inducing the state of myosin ADP·Pi (ADP, Adenosine diphosphate; Pi, Phosphoric acid) held together by weak interactions with F-actin (Franz et al., 2020). The weak binding state of actin·myosin·ADP·Pi is sensitive of Ca^2+^, and much easier to transform into a strong binding force state. Increased weak binding state accelerates the rate of force generation, as well as changing the permeability of cell gap. Phosphorylation of myosin-light-chain kinase could induce the involvement of related antibodies to decrease blood-brain barrier permeability (Liu et al., 2018). The degradation of myosin light chain 2 activated cellular apoptosis in Alzheimer’s and Parkinson’s disease (Guo et al., 2019; Wang et al., 2019). Myosin light chain kinase (MLCK) activates the JNK (c-Jun N-terminal kinase) signaling pathway to facilitate neuronal apoptosis in high glucose-induced hippocampal neurons (Xu et al., 2021) (Figure 3).

#### 2.2.3 Cell of the immune system adhesion and migration

**FIGURE 4:**
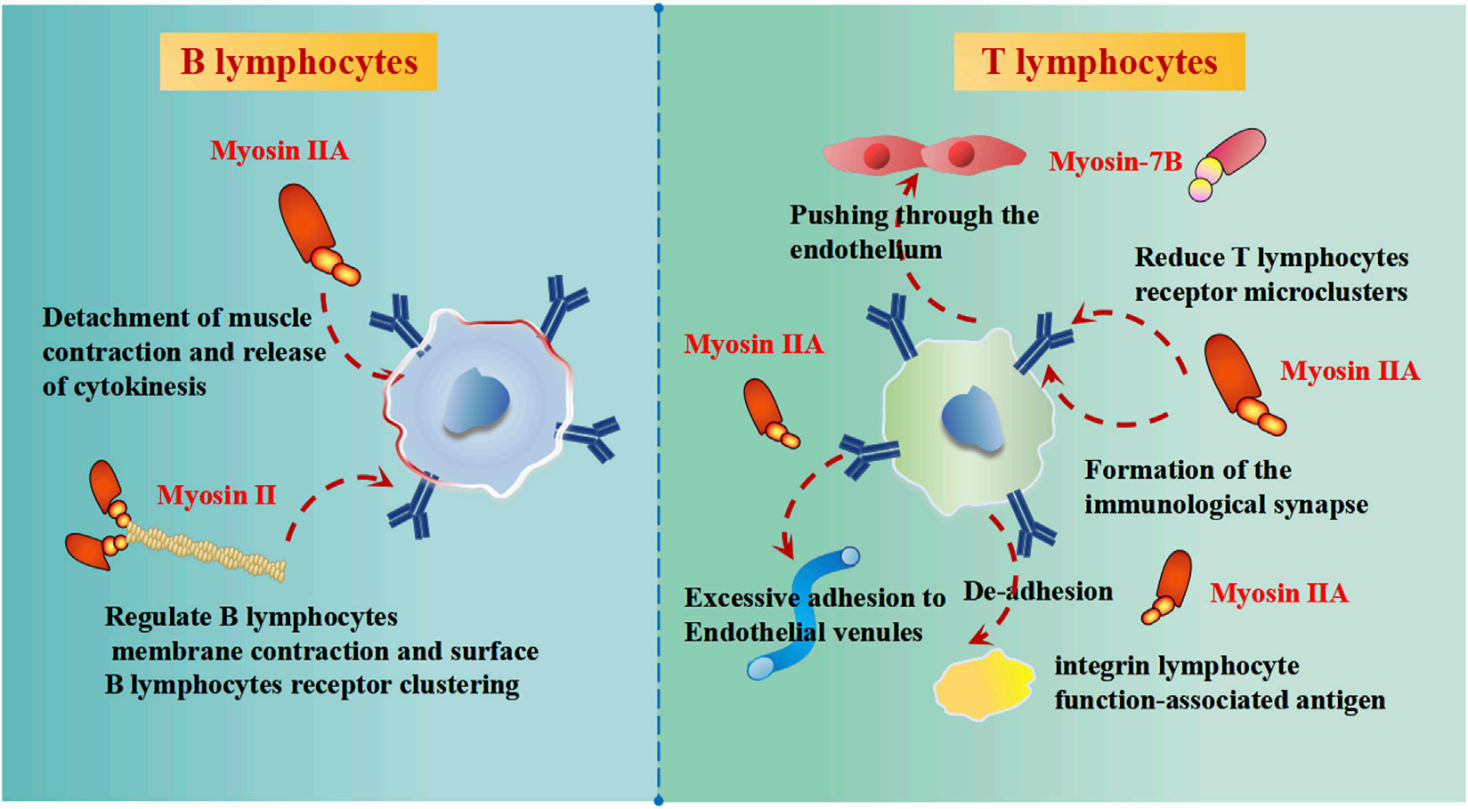
The effects of myosins on B lymphocytes and T lymphocytes of immune function.

Cell of the immune system adhesion and migration are critical for immune response and homeostasis. The dysregulation of cell microenvironments triggers aberrant cell of the immune system adhesion and migration. New research on myosin suggests that myosins participate in the process of adhesion and migration. Myosin-7B (MYO7B) could regulate the cell-to-cell transmission in Parkinson’s disease (Zhang et al., 2020b). Non-muscle myosin II is essential for the formation of the B cell receptor surface, influencing the signal transmission to antigen-presenting cell (Seeley-Fallen et al., 2022). T lymphocytes lacking myosin IIA showed excessive adherence to high endothelial venules, reduced migration in the interstitium, and inefficient recirculation in the lymph nodes (Jacobelli et al., 2010) (Figure 4). The function of myosin IIA can also be observed by its participation in immune synaptic maturation (Kumari et al., 2012), mediated primarily by regulating lymphocyte function-associated antigen-1 to promote the mechanical force change involving detachments and contractions (Morin et al., 2008) (Figure 4). According to the patho-anatomy of the disease, the phenomenon of mst1 deficiency involves overactivated T lymphocytes and abnormally differentiated B lymphocytes (Jacobelli et al., 2010). T lymphocytes migrate through the transcellular process during interstitial migration, and myosin IIA is the driving force for pushing the nucleus through the endothelium (Krummel et al., 2014) (Figure 4). Lamin-A enhances the extent of phosphorylation of myosin IIA and Vav1 (Vav guanine nucleotide exchange factor 1), which could lead to increased activation of the T lymphocytes receptor, accelerating formation of the immunological synapse between T lymphocytes and antigen-presenting cells (González-Granado et al., 2014) (Figure 4).

### 2.3 Abnormal expression of myosins in autoimmune diseases

#### 2.3.1 Multiple sclerosis

In a specific type, ALS-associated (ALS, Amyotrophic lateral sclerosis) extraocular muscles express a unique set of myosin isoforms and dampen multiply innervated fiber contraction (Nijssen et al., 2017) (Table 1). Evidence continues to accumulate associating myosin evidences with multiple sclerosis. Unconventional myosin 48/Myo1C (Myosin 1c) is responsible for the progression of pathological ALS diseases, while myosin 48/Myo1C interacts with other proteins to participate in the complement and coagulation cascades of ALS diseases (Myronovkij et al., 2016; Starykovych et al., 2021). An isolated 46 kDa form of unconventional Myo1C present in MS (Multiple sclerosis) patients via specific autoantibodies can serve as a potential screening protein (Zasońska et al., 2018). Thus far, monospecific anti-p46/Myo1C immunoglobulin G (IgG) antibodies might be expected to have a wide potential application in areas in earlier age at MS disease diagnosis (Horák et al., 2017).

**TABLE 1:** Myosin and its physiological function in autoimmune diseases.

Myosin	Physiological function
α-myosin peptides	Expand peripheral blood T lymphocytes in diseased heart and skeletal muscle; As the candidate autoantigens in myocarditis
Non-muscle myosin	As the immunopathology factor inducing neuroinflammation
Myo1C and myosin 48	Participate in the complement and coagulation cascades
MYO5b	Cause cholestasis with normal serum gamma-glutamyl transferase activity
MYO18A	Induce mixed features of myelodysplastic/myeloproliferative neoplasms in myeloid leukemia patients
Myosin heavy chain alpha	Trigger CD4^+^ T lymphocytes accumulating in the myocardium
MYH6	As myocardial antigen in myocarditis
MYH9	Induce the increased expression of α-synuclein
Myosin heavy chain 11	Impair mitotic progression
Embryonic myosin heavy chains	Weaken the contractile force of gastric circular smooth muscle and gastric myoelectric activity
Myosin light chain 1	Influence dopamine neuronal differentiation
Myosin light chain 20	Impair mitotic progression
α-smooth muscle actinmyosin Ⅱ	Impair mitotic progression
FLNA regulated the actin-myosin motor units and cytoskeleton	Fail to transmit force signals to the extracellular matrix

#### 2.3.2 Alzheimer’s and Parkinson’s disease

Transcriptome changes involving a complex set of cytoskeletal proteins, including nuclear lamin, tropomyosin 1, and myosin light chain 1, were specifically upregulated during Parkinson’s disease pathogenesis (Ryu et al., 2019). *Pink1* ^*−/−*^ mice (Parkinson’s disease model) showed enhanced tongue press force with relative increases in myosin heavy chain IIa in the styloglossus but typical myosin heavy chain profiles in the genioglossus (Glass et al., 2020). Cortical myosin II damage is affected in the metaphase plate in cell mitosis in associated Parkinson’s diseases (Toyoda et al., 2017). The abnormal expression of myosin light chain 20, myosin heavy chain 11, and α-smooth muscle actin participated in gastroparesis of Parkinson’s disease (Xiu et al., 2020).

#### 2.3.3 Other neurological disorders

There are many kinds of autoimmune diseases that occur in the nervous system. Even for some common nervous system diseases, the inclusion of a new classification as part of autoimmune diseases is needed. Except for multiple sclerosis, Alzheimer’s and Parkinson’s disease, with the development of newly discovered molecular mechanisms, autoimmune diseases are increasing. Embryonic myosin heavy chains are markers that are more commonly expressed in spinal muscular atrophy (Sewry et al., 2021). The uncovered CD8^+^ T cell response activated by T lymphocytes simulates the effects of perturbing non-muscle myosin and vimentin structure, which suggests that myosin is an important immunopathology factor in neuroinflammation in experimental autoimmune encephalomyelitis (Feizi et al., 2021).

#### 2.3.4 Autoimmune myocarditis

Except the myosin immunization functions on autoimmune myocarditis, various myosin subtypes have been found for autoimmune myocarditis. α-Myosin peptides expand peripheral blood T lymphocytes in diseased heart and skeletal muscle and might be candidate autoantigens in myocarditis (Axelrod et al., 2022). Myosin heavy chain alpha (MYHCA)-triggered post-MI CD4^+^ T lymphocytes selectively accumulate in the myocardium of infarction mice as the dominant cardiac antigen (Rieckmann et al., 2019).

#### 2.3.5 Myositis

The main inducement of heart failure and sudden cardiac death in young adults and adolescents is myocarditis. Cardiac myosin, as the major autoantigen, is the critical cause associated with autoimmune processes in myocarditis clinical cases. In some cases, architectural abnormalities in muscle myosin were detected in congenital myopathies via muscle biopsy (Claeys, 2020). Many new genes are being identified in congenital myopathies specializing in neuromuscular disorders. Recently, a patient with a homozygous mutation presented a dominant phenotype of rare muscle disorders, while MYH2 (Myosin heavy chain 2) mutations were responsible for autosomal dominant progressive myopathy (Telese et al., 2020). Prune belly syndrome (PBS) is a rare, multisystem congenital myopathy characterized by dysfunction of the X-chromosome gene filamin A (FLNA) in transmitting force signals from the actin-myosin motor units and cytoskeleton to the extracellular matrix (Iqbal et al., 2020). MYH7 is an extremely rare gene that provides novel insights into associated changes in muscle physiology (Beecroft et al., 2019). Additionally, uncoordinated mutant myosin chaperone 45B (UNC45B), as the default template assembly, splits up the muscle thick filament arrangement (Dafsari et al., 2019).

#### 2.3.6 Hemopathy

Thrombocytopenia is characterized by a decrease in platelets or abnormal platelet function, accompanied by clinical presentations such as menorrhagia and ecchymosis and other clinical manifestations such as neuropathic deafness, cataracts, and nephritis. Thrombocytopenia, an immune disorder, is a new hot topic. There appear to be four main causes of autoimmune thrombocytopenia: 1) the production of anti-platelet autoantibodies, which are mainly produced in the spleen and destroy platelets; 2) platelet-reactive T cell abnormalities in the blood of patients with immune thrombocytopenia; 3) the body’s immune response to platelet-associated antigens; 4) viruses that cause immune complex disease resulting in thrombocytopenia. Molecular mechanisms directing hereditary thrombocytopenia are often related to gene mutations. Dohle inclusions were more likely to be found in areas of peripheral blood mutant neutrophils in MYH9-mutation (MYH9, Non-muscle myosin heavy chain 9) hereditary thrombocytopenia (MYH9-related diseases are caused by defects in the gene encoding myosin heavy chain MYH9) (Balduini et al., 2018; Rodeghiero et al., 2018). A novel variant (E1421K) induced abnormal aggregates of MYH9 protein manifested as apparent neonatal alloimmune thrombocytopenia MYH9 (Samelson-Jones et al., 2018). The essential fusion of FLT3 (Fms Related Receptor Tyrosine Kinase 3) and MYO18A (Myosin 18 alpha) was detected in eosinophilic granulocytes associated with chronic myeloproliferative disorders, such as atypical chronic myeloid leukemia (Zhang et al., 2018).

#### 2.3.7 Inclusion body disease

Inclusion body disease (IBD) has 90% of its origins in inclusion body herpesvirus. The virus produces inclusion bodies in respiratory and digestive tract epithelial cells, developing secondary bacterial infection caused by CD8^+^ lymphocyte-mediated autoimmune cytotoxicity. Abnormal changes in retinal tissues, brain tissues, spinal cord tissues, terminal nerve tissues, and organ tissues are common complications of IBD, and IBD during onset is hidden, has a slow progression, and has a longer course. To date, there is still no specific drug treatment. The main clinical manifestations of microvillus inclusion disease (MVID) are refractory diseases accompanied by severe watery diarrhea, failure to thrive, and metabolic acidosis. Newly discovered mutations in the myosin Vb (MYO5B) gene have been identified as causative factor for microvillus inclusion diseases (Gonzales et al., 2017). MYO5B deficiency produced a pathological process of isolated cholestasis (Cartón-García et al., 2015). Likewise, another study has the same implications as previous studies; MYO5B (myosin 5B) mutation-induced microvillus inclusion disease (MVID) causes fatal autosomal recessive congenital diarrheal disorders (Dhekne et al., 2018).

### 2.4 The participation mechanism of myosins in the pathophysiology of autoimmune disorders

#### 2.4.1 Molecular mechanisms of myosins in the central nervous system

In response to the pathological conditions, the physiological shape of axons is altered in several diseases, such as Alzheimer’s and Parkinson’s diseases. The contraction dysfunction via myosin motor is one of the significant reasons that lead to the altered axons morphology (Riccobelli, 2021). Additionally, different types of myosin can be found in particular conformations. Galectin-3 (MAC-2) in microglia dynamically adjusts the state of actin/myosin-based contractions to lead to the retraction of filopodia/lamellipodia to damage myelin surrounding the central nervous system axons in multiple sclerosis (Reichert and Rotshenker, 2019). Phosphorylation of myosin-light-chain kinase was moved to the inactivated state under treatment with an A2A (Adenosine A2A Receptor) receptor agonist, which could decrease blood-brain barrier permeability in multiple sclerosis disease A2A (Liu et al., 2018).

In recent years, it has been suggested that Alzheimer’s disease might not only be a neurological disease but also an autoimmune disease. Meanwhile, research on Parkinson’s disease and its potential association with autoimmune diseases is strongly being pursued. Autoimmune mechanisms include inflammatory factors, the Golgi network, and gene mutations. TNF (Tumor Necrosis Factor)-α elevated inflammatory factor expression induced hippocampal neuron downregulation while accelerating the degradation of myosin light chain 2-activated caspase-3 activation and apoptosis in Alzheimer’s and Parkinson’s disease (Guo et al., 2019). The dysfunction of myosin proteins is a crucial intracellular process for the pathogenesis of Alzheimer’s and Parkinson’s diseases. Research has shown that all myosin Myo1 and 2 retrieved from the endosome and the vacuole participate in the recycling traffic of the trans-Golgi network (Nguyen et al., 2021). An alternative theory is that the activation of LRRK2 (Point mutations in leucine-rich repeat kinase 2) kinase caused the combination of RILPL2 (Rilp-like proteins 2) and phosphorylated LRRK2 to interfere with myosin Va’s role in ciliogenesis (Dhekne et al., 2021). Tau depends on myosin ATPase activity and reduces the phosphorylation of regulatory myosin light chains in Alzheimer’s disease Tau (Wang et al., 2019). Myosin monomers can aggregate rod-like morphology, including liposomes and viruses, by interacting with α-synuclein in Alzheimer’s and Parkinson’s diseases (Glass et al., 2019).

Diabetic encephalopathy, the most common form of type 1 diabetes, is an autoimmune disease and immune-mediated form of neurological disorder. In this disease, myosin light chain kinase (MLCK) activates the JNK signaling pathway to facilitate neuronal apoptosis in high glucose-induced hippocampal neurons (Xu et al., 2021). The pathogenesis of diabetic encephalopathy is caused by autoimmunity, mainly the regulatory JNK/MLCK signaling pathway (Xu et al., 2021).

#### 2.4.2 Molecular mechanisms of myosin in the peripheral nervous system

There are other nerve symptoms, such as sublingual muscle weakness and abnormal gastric motility, caused by Parkinson’s disease. More research associated with Parkinson’s disease pathogenesis. Neuromuscular pathology found that MyHC2L was associated with muscle fiber abnormalities in the larynx and pharynx in patients with Parkinson’s disease (Hoover and Murphy, 2020). Myosin-7B (MYO7B) is a critical endocytosis regulator in the cell-to-cell transmission of misfolded α-synuclein in Parkinson’s disease (Zhang et al., 2020b).

Additionally, diseases in the peripheral nervous system, such as Guillain‒Barre syndrome and myasthenia gravis, are additional autoimmune diseases caused by damage to the neuromuscular junction. Charcot-Marie-Tooth disease (CMT), also called hereditary motor and sensory neuropathy (HMSN), is a common peripheral nerve monogenetic disease. CMT disease is characterized by gradual onset and slow progressive weakness and atrophy of distal limb muscles and other features of impaired sensation. Myelin synthesis and actinomycin dysfunction are the basis of abnormal spinal cord function in Charcot-Marie-Tooth disease. Charcot-Marie-Tooth type 4B1 (CMT4B1) is a severe autosomal recessive demyelinating neuropathy with childhood onset caused by loss-of-function mutations in the myotubularin-related 2 (MTMR2) gene. MTMR2 mediates mTORC1-dependent myelin synthesis and RhoA (Ras Homolog Family Member A)/myosin II-dependent cytoskeletal dynamics to influence myelin membrane expansion and longitudinal myelin growth Charcot-Marie-Tooth 4B1 (CMT4B1) (Guerrero-Valero et al., 2021).

The main inducement of heart failure and sudden cardiac death in young adults and adolescents is myocarditis. Cardiac myosin, as the major autoantigen, is the proegumenal cause associated with autoimmune processes in myocarditis clinical cases. In a mouse model, cardiac myosin is an autoantigen, especially in the later phases of postinfectious myocarditis. Heart-reactive antibodies were identified as cardiac isoforms of myosin due to autoimmunization (Rose et al., 1987). The application of emulsified cardiac myosin itself could establish an experimental autoimmune myocarditis model (Lee et al., 2016). Cardiac myosin could stimulate the production of heart-specific antibodies accompanied by cardiac muscle striations and sarcolemma (Rose et al., 1987). In cardiac myosin-immunized experimental autoimmune myocarditis mice, activated extrathymic T lymphocytes expressed high levels of LFA-1 (Lymphocyte function-associated antigen 1) and IL-2R beta-chains while inducing differentiated CD4-CD8- T cell movement to the sites of the cardiac lesion (Hanawa et al., 1993). Myocarditis depends on cardiac myosin heavy chain 6-specific T helper TH17 cells imprinted in the intestine by a commensal *bacteroides* species peptide mimic (Gil-Cruz et al., 2019).

#### 2.4.3 Molecular mechanisms of myosins in the muscle

Autoimmune polymyositis is an extensive inflammatory lesion in muscles. The clinical manifestation usually presents with myasthenia, accompanied by muscle pain, muscle atrophy involving difficulty swallowing, or breathing, in terms of elevated circulating levels of inflammatory markers. Congenital myasthenia syndromes are a group of rare, inherited disorders characterized by impaired function of the neuromuscular junction. MYO9A mutant zebrafish were used as a quality model for studying congenital myasthenic syndromes. MYO9A might impair the function of neuromuscular junctions during embryonic development (O’Connor et al., 2018). Drug therapy is an important adjunct to immunological treatments. The novel agent of 3-n-butylphthalide (NBP) associated with autoimmunity of the drug target was used for the treatment of myositis and other muscular diseases (Chen et al., 2018).

## 3 Discussion

Inbred strain mice are mainly chosen by laboratory due to the stable gene homozygous, consisten phenotype, clear background. The genetic background of Balb/C and C57BL/6J mice were used in the autoimmune modeling experiment commonly. C57BL/6 is the preferred genetic background for diet-induced obesity, multiple sclerosis models, or chronic experimental autoimmune encephalomyelitis. Balb/C mice are the most commonly used animals in the research fields of tumor, inflammation, and autoimmunity, while almost all of the mice derived myeloma cells for cell fusion were obtained from Balb/C mice. Balb/C and C57BL/6J mice have differences in the aspects of Th1 and Th2 immune responses. Under the infection and allergic irritation, C57BL/6 mice are dominated by Th1 immune response and IFNγ, while Th2 immune responses were easier to be triggered in Balb/C mice. Balb/C mice tend to own the humoral response on a larger scale than that in C57BL/6 mice (Zamora et al., 2021).

The human major histocompatibility (HLA) complex is a group of closely linked genes located on the broken arm of human chromosome 6. The autoimmune patients have a higher incidence rate of genetic propensity, while HLA antigen is the most known genetic risk factor for autoimmune diseases. On one hand, HLA antigen offers the toxins or excessive pro-inflammatory factors to body system (Bodis et al., 2018). On the other hand, HLA gene polymorphisms might destroy the autoimmune state, such as HLA-DQ2 and HLA-DQ8 related with celiac diseases, HLA-DRB1 related with rheumatoid arthritis, and HLA-b27 associated with spondyloarthritis (Vandenbroeck, 2012). As the parameters used in clinical testing, cardiac antigens and HLA expression were used in the diagnosis of myocarditis (Rose, 2009).

Cardiac-specific protein α-myosin, though absent from the thymus, could expand peripheral blood T cells, indicating it might be a clinically important autoantigen in fulminant myocarditis (Axelrod et al., 2022). For regulatory T cells dominance, under non-inflammatory conditions, T cells specific for myocardial antigens specific associated α-myosin heavy chain peptides differentiated into expanded clones of regulatory T cells. Under the cardiac infection and/or genetic variations in peripheral tolerance, effector T cell derived cytokines inhibit expansion of regulatory T cells that contributes to the inflammatory damage to the heart in autoimmune myocarditis (Lichtman, 2013). Transgenic expression of α-MYHC (such as MYH6) in thymic epithelium conferred tolerance to cardiac myosin and prevented myocarditis, demonstrating that α-MYHC is a primary autoantigen in mediating central and peripheral T cell tolerance. For example: MYH6 is suggested to be targeted on CD4^+^ T cell in a spontaneous mouse model of myocarditis (Lv et al., 2011).

In cases with severe complications, large deposits of anti-tropo-myosin antibody were most commonly found on the blood vessel wall. This autoimmunity is also controlled by many factors, which are affected by intra- or extra-cellular factors. Internal factors include intracellular calcium, ATP, endogenous enzymes, and related proteins. Internal Factor 1: calcium ion. 1) Intracellular calcium is an important element of the weakening and destruction of the complete structure of myofibrils. The elevation of calcium directly or indirectly induced the combination of actin and myosin to form actin-myosin protein (Awinda et al., 2022). Two theories exist to explain the reason. One is that when calpain is activated, the action could spur the dissociation of myofibrillar protein (Koohmaraie et al., 1989). The other is the calcium ion theory (Koohmaraie et al., 1989). When calpain declines, calcium ions alone lead to further collapse and breakage in muscle fibers. This weakened myofibrillar skeleton is formed by calcium ions rather than calpain (Berchtold et al., 2000). Some studies have found a beneficial association between increased acculturation and mental health, whereas others have found a detrimental association or no relationship at all. Other studies tend to study the activation of calpain, primarily in terms of calcium ions, and activated calpain promotes the degradation of myofibrillar skeletal proteins, directly or indirectly influencing the connection of actomyosin transverse bridges (Geesink et al., 2001).

Internal Factor 2: ATP. The sustainable consumption of ATP rapidly causes an increase in hydrolysates such as AMP (Adenosine monophosphate) and IMP (Inosinic acid) (Okitani et al., 2008). Only ADP, calcium ions, or ATP have been ineffective in the dissociation of actomyosin (Benjakul et al., 2007). However, IMP, AMP, and the high concentration of phosphate radical (PO_4_^3-^) facilitated the dissociation of actomyosin, which was irreversible. Internal Factor 3: endogenous enzyme. Among the various endogenous enzymes, the three main proteins include calpsin, lysosomal protease (cathepsin), and proteasome. Calpsin is a calcium-activated protease, and there are two isoforms of calpsin, μ-calpsin and m-calpsin, which are very similar in their structure but are very different in their function within organisms. Released amounts of Ca^2+^ activate μ-calpsin to induce the degradation of actin, myosin, and troponin in myofibrillar fibers (Tatsumi et al., 1993). Cathepsin would have been in the form of a proenzymer, and cathepsin further reduced the proteins based on the degradation of calpsin (Dutaud et al., 2006). The proteasome showed a similar effect as cathepsin to further reduce the proteins (Sentandreu et al., 2002).

Other potential external factors include ions, high pressure, and phosphorylation. The combined action of MgCl^2^ and pyrophosphate PO_4_^3−^ triggers the natural dissociation of actin myosin. High-pressure treatment changes the osmotic pressure of cells, resulting in the rupturing of cell walls so that a variety of enzymes in the lysosome are released, which leads to an increased degree of hydrolysis of myosin and actin, affecting the dissociation of actomyosin. Myosin light chain phosphorylation is a complicated process (Lee et al., 2022) and is influenced by many factors, including Ca^2+^ concentration, calmodulin, myosin light chain kinase, and protein kinase A and C. Myosin light chain phosphorylation promotes myosin regulated light chain phosphorylation (Anis et al., 2022) or inhibits myosin light chain phosphorylase activity through activation of Rho kinase. Thus, they did see some increase in myosin regulated light chain phosphorylation, leading to muscle contraction or sarcolemma migration (Hirano and Hirano, 2022; Lei et al., 2022).

The most immediate response is inflammation at the site of autoimmune diseases and the recruitment of additional cells of the immune system, including neutrophils. Tissue deformation, necrosis, infiltration, and fibrosis cause the body to release proinflammatory cytokines and immune factors that initiate autoimmune responses (D’Amico et al., 2022). This pathological progress weakens the immune response and can lead to organ inflammation and damage. Otherwise, cytokines are immune system signaling chemicals, and their production is a first step in causing inflammation (Zhan et al., 2021). Autoimmune diseases are believed to be connected to an inflammatory process initiated by the body’s immune response.

At present, improvement in more targeted biological agents has spring up, while a serial of effective biological medicine were invented, including Belimumab, Sirukumab, Rituximab, Tocilizumab, Infliximab, Etanercept, Etc. Also, the symptoms of myasthenia could be alleviated by drugs on myosin immunized experimental autoimmune myositis model, like butylphthalide, while the mechanism of butylphthalide is based on the increase of Ca^2+^-ATPase activities of muscle mitochondrial membrane and the decrease of IFN-γ (Interferon-γ) mRNA expression (Chen et al., 2018). In 2021, N Engl J Med published that Omecamtiv (a novel myosin agonist), significantly improved composite cardiovascular endpoints in patients with heart failure with reduced ejection fraction (Bellumkonda et al., 2021). In 2022, JAMA confirmed that Omecamtiv significantly improved the capacity of exercise in heart failure with reduced ejection fraction patients, that other anti-heart failure drugs do not get with similar characteristics (Lewis et al., 2022). In addition, there have been numerous development of myosin targeted drugs, such as Mavacamten capsules (the first oral heart-specific allosteric inhibitor of cardiac myosin, treatment for hypertrophic cardiomyopathy, which reduced the ATPase activity of cardiac myosin heavy chain, inhibited myocardial overcontraction, and increased diastolic compliance); Aficamten (CK-3773274) (a cardiac myosin inhibitor, treatment for hypertrophic cardiomyopathy, which directly bound to cardiac myosin, reduced myocardial contractility and excessive contraction). The new findings published in the 2022 Nature could help researchers to design new myosin-related drugs that overcome autoimmune diseases. The T lymphocytes were sequencing to rebuild their receptors, specific peptides of alpha myosin were determined in T lymphocytes based on the receptors. There have been demonstrated that specific peptides interestingly were found in T lymphocytes in myocarditis patients, while T lymphocytes from patients have the same source of antigen (Axelrod et al., 2022). In the future, scientists expect to gain more experience from the specific myosin related antigen and develop more specific targeted drugs for years to come.

## 4 Conclusion

In conclusion, it is particularly important to understand myosin-related autoimmune diseases. Myosins are involved in autoimmune diseases, and take part in the pathological process of immune responses. Additionally, myosin dysfunction causes dystonic cramps, abnormal muscle contractions, and chronic energy deficiency that can lead to severe symptoms presented by autoimmune diseases. Although research on the role of myosin in the occurrence and development of autoimmune diseases is still in its infancy, it is of great clinical relevance, and its mechanism remain to be further studied.

## Figures and Tables

**Table:** 

**α-MYHC**	Alpha myosin heavy chain
**A2A**	Adenosine A2A Receptor
**AD**	Autosomal dominant
**ADP**	Adenosine diphosphate
**ALS**	Amyotrophic lateral sclerosis
**AMP**	Adenosine monophosphate
**ATP**	Triphosadenine
**CaM**	Calmodulin
**CMT**	Charcot-Marie-Tooth Disease
**CMT4B1**	Charcot-Marie-Tooth type 4B1
**FLNA**	Filamin A
**FLT3**	Fms Related Receptor Tyrosine Kinase 3
**HLA**	Human major histocompatibility
**HMSN**	Hereditary motor and sensory neuropathy
**IBD**	Inclusion body disease
**IFNγ**	Interferon gamma
**IgG**	Immunoglobulin G
**IMP**	Inosinic acid
**IL-2R**	Interleukin 2 receptor
**JNK**	c-Jun N-terminal kinase
**LFA-1**	Lymphocyte function-associated antigen 1
**LRRK2**	Point mutations in leucine-rich repeat kinase 2
**MLC2**	Regulatory light chain 2
**MLCK**	Myosin light chain kinase
**MS**	Multiple sclerosis
**MTMR**2	Myotubularin-related 2
**MVID**	Microvillus inclusion disease
**MYH2**	Myosin Heavy Chain 2
**MYH6**	Myosin Heavy Chain 6
**MYH9**	Non-muscle myosin heavy chain 9
**MYHCA**	Myosin heavy chain alpha
**Myo1C**	Myosin 1c
**MYO5B**	Myosin Vb
**MYO7B**	Myosin-7B
**MYO18A**	Myosin 18 alpha
**NBP**	3-n-butylphthalide
**PBS**	Prune belly syndrome
**Pi**	Phosphoric acid
**PO_4_^3-^**	Phosphate radical
**Rilpl2**	Rilp-like proteins 2
**RhoA**	Ras Homolog Family Member A
**TNF**	Tumor necrosis factor
**UNC45B**	Uncoordinated mutant myosin chaperone 45B
**Vav1**	Vav guanine nucleotide exchange factor 1
**IFN-γ**	Interferon-γ
