# Supplementary material for: Background and roles: myosin in autoimmune diseases
Source: Front Cell Dev Biol. 2023 Aug 23;11:1220672. doi: 10.3389/fcell.2023.1220672 (PMC10484797; doi:10.3389/fcell.2023.1220672)
Supplement: Supplementary file 1 [file Table1.DOC]

**Table 1 Abnormal Expression of Myosins in Autoimmune Diseases**

| **Autoimmune diseases** | **Sample source** | **Detection technology** | **Myosin** |
| --- | --- | --- | --- |
| Multiple Sclerosis | blood serum of patients | MALDI TOF/TOF mass-spectrometry | myosin 48/Myo1C ^[34-35]^ |
|  | blood of patients | Monodisperse magnetic poly(glycidyl methacrylate) microspheres | 46 kDa form of unconventional Myo1C ^[36]^ |
|  | blood of patients | Magnetic poly(2-hydroxyethyl methacrylate) microspheres | 46 kDa form of unconventional Myo1C ^[37]^ |
| Alzheimer's and Parkinson's disease | dopamine neurons | proteomic analysis | myosin light chain 1 ^[38]^ |
|  | tongue tissues of Pink1^-/-^ mice | western blot | myosin heavy chain Ⅱa ^[39]^ |
|  | mitotic cells | Genome-scale single-cell omics | myosin heavy chain 11, and α-smooth muscle actinmyosin Ⅱ ^[40]^ |
|  | blood of patients (gastroparesis in Parkinson's disease) | western blot | myosin light chain 20, myosin heavy chain 11 ^[41]^. |
| Spinal muscular atrophy | muscle of patients | muscle biopsy | Embryonic myosin heavy chains ^[42]^ |
| Autoimmune encephalomyelitis | experimental autoimmune encephalomyelitis mice | immunization approaches, multiparametric flow cytometry, and functional assays | nonmuscle myosin and vimentin ^[43]^ |
| Autoimmune myocarditis | peripheral blood T lymphocytes from diseased heart and skeletal muscle of patients | single-cell RNA and T lymphocytes receptor (TCR) sequencing | α-myosin peptides ^[44]^ |
|  | transgenic murine cardiac myosin-specific CD4+ T lymphocytes of patients | transcriptomic | myosin heavy chain alpha ^[45]^. |
| Myositis | muscle of patients | muscle biopsy | myosin (myosin storage myopathy) ^[46]^ |
|  | muscle of patients | muscle biopsy and NGS followed with sanger sequencing | MYH2 ^[47]^ |
|  | blood or saliva of patients | whole exome sequencing | FLNA regulated the actin-myosin motor units and cytoskeleton ^[48]^ |
|  | muscle of patients | muscle biopsy and gene sequencing | MYH7 ^[49]^ |
|  | muscle of patients | muscle biopsy and whole exome sequencing | mutant myosin chaperone 45B (UNC45B) ^[50]^ |
| Hemopathy | blood of patients | immunofluorescence assay | myh9 ^[53]^ |
|  | blood of patients (chronic myeloproliferative disorders) | cytogenetics and Fluorescence In Situ Hybridization | MYO18A ^[54]^ |
| Inclusion body diseases | blood of patients | gene sequencing | myosin Vb (MYO5B) ^[55]^ |
|  | duodenal samples of Myo5b knockout mice | histology and immunohistochemistry | Myo5b ^[56]^. |
|  | blood of patients | gene sequencing | myosin Vb (MYO5B) ^[57]^ |
